# Organisational Commitment in Healthcare Systems: A Bibliometric Analysis

**DOI:** 10.3390/ijerph18052271

**Published:** 2021-02-25

**Authors:** Carlos de las Heras-Rosas, Juan Herrera, Mercedes Rodríguez-Fernández

**Affiliations:** Department of Economics and Business Administration, Universidad de Málaga, 29071 Málaga, Spain

**Keywords:** organisational commitment, healthcare, bibliometric, job satisfaction, SciMAT

## Abstract

Business organisations are subject to high pressure to ensure their sustainability and competitiveness. In the case of healthcare institutions, moreover, there are unique characteristics where human resource management is of vital importance. The workforce in these institutions is at a critical moment where the shortages of qualified staff, burnout, or job dissatisfaction represent some of the detrimental aspects for the performance of the organisation, and more importantly, they diminish the quality of patient care. The promotion of organisational commitment is positioned as one of the tools that organisations have to face this problem. This paper aims to increase knowledge about research trends that analyse organisational commitment in healthcare institutions. To this end, using bibliometric techniques, a sample of 448 publications on this subject from journals indexed in Web of Science between 1992 and 2020 is analysed. The results obtained suggest a growing interest in this subject and a visible concern for the management of human resources in these institutions. Research has focussed mainly on organisational factors related to nursing staff. The most analysed topics have been job satisfaction, the implications of stress and high turnover, burnout syndrome, and the possibility of leaving the job. On the other hand, issues emerged such as empowerment in the workplace and others related to organisational management such as quality of service or performance. Finally, there is a lack of research that deals more deeply with other groups working in health centres, such as doctors or administrative staff. There is also a need for further development in the analysis of the implications of the ideological psychological contract in relation to normative organisational commitment in the field of healthcare organisations. The contribution of this work focusses on expanding knowledge about commitment in healthcare organisations and creating points of support for future research as well as helping healthcare managers make decisions in HR management.

## 1. Introduction

In recent times, healthcare delivery systems face a continuous challenge brought about by a number of factors. These include continuing technological change, clinical advances, and increasing societal expectations of such organisations [1,2]. Managers and leaders of healthcare facilities are faced with an organisational model that, in addition to the usual values of effectiveness and efficiency, must meet excellent standards of clinical care [3]. If in general, business organisations need to have professionals who are aligned with the strategic objectives of the company and demonstrate a clear orientation towards work and organisation, in the field of health, human resources management takes on an even more important role, since most of these employees are the ones who interact with patients on a daily basis, and quality and medical care are at stake [4,5].

Numerous studies have addressed issues directly related to employees in the health sector. Studies have shown that there is a higher risk of burnout among healthcare employees [6,7,8], and high staff turnover in healthcare facilities has been the subject of much research analysing its causes [9,10,11,12]. The intention to leave the job or even the profession on the part of health personnel remains a major concern for health centre administrators, and this aspect has also been addressed by researchers, more specifically in the case of nursing personnel [13,14,15]. Another health sector-related element addressed in the analyses is the insufficient availability of qualified nursing staff. This has been noted in European countries [16], the USA [17], Canada, the UK, and Australia [18], among others. This shortage of staff may be due to various factors related to working conditions, professional recognition, or even lack of motivation. The World Health Organisation, in its 2006 World Health Report [19], already warned of the worrying staff shortages and problems faced by hospitals and health centres worldwide. The shortage of nursing staff will have a number of implications for employers. The average age of nursing staff is increasing [20], and this means that these older and more experienced workers will require better working conditions, which together with the aforementioned shortage of qualified staff will lead to higher wages in this sector [20]. Needleman et al. [21] indicate that prolonged inadequate hospital nurse staffing can increase operational and labour costs and also lead to an increased likelihood of adverse patient outcomes.

It seems clear that the health sector is currently in a complex situation with regard to the management of human resources in its workplaces. Proposals and actions taken to minimise problems of staff turnover, attrition, shortages, productivity, job satisfaction, and organisational commitment are varied and have been the subject of research. Some studies have focussed on the leadership styles of managers and leaders, where different behaviours and strategies for empowering staff have been analysed [22,23,24]. The connection between the psychological contract and organisational and professional commitment has also been addressed by researchers [25,26,27]. Organisational commitment has been notoriously linked in the scientific literature with the fulfilment of the psychological contract [28,29]; the validity or degree of fulfilment of the psychological contract is a key aspect in the behaviour, attitude, and performance of employees. Non-compliance with the psychological contract generates less commitment on the part of employees, which can manifest itself in lower job performance [30], greater intention to leave [31], lower job satisfaction [32,33], and less trust in the organisation [34]. The fulfilment or non-fulfilment of obligations between employee and employer greatly affects the organisational commitment that the employee develops. The management of human resources in any organisation, and particularly in health institutions, is one of the main functions that must be developed and managed appropriately by those in charge. Proper analysis of working conditions, employee well-being, and job satisfaction will provide high levels of organisational commitment [35,36].

We understand that the analysis of organisational commitment developed by employees is a key tool in organisations of any kind. Healthcare institutions present themselves as particular cases where organisational commitment takes on even greater importance. These healthcare organisations show particular characteristics in their workplaces, mainly in front-line healthcare staff [7,37], where the following can be highlighted: prolonged work stress; physical and emotional exhaustion; direct contact with the patient; and the uncertainty of the workload according to shifts or the ethical work climate. This is followed by an exploration of the concept of organisational commitment and other data related to health centres.

### 1.1. Workers in Healthcare Facilities

The International Standard Classification of Occupations (ISCO-08) [38] distinguishes health professionals in Major Group 2, Sub-major Group 22. Within this Major Subgroup 22, there are Subgroups: 221 Physicians; 222 Nursing and midwifery professionals; 223 Traditional and alternative medicine professionals; 224 Paramedical practitioners; 225 Veterinarians; and 226 Other health professionals. From here, a number of primary groups are classified to describe specialties that are not the subject of this analysis. The World Health Organization (WHO) report “World Nursing Status 2020” [39] indicates that among all these occupational groups related to the health professions, approximately 59% correspond to Major Subgroup 222, Nursing and midwifery professionals. Nursing represents the largest group at the structural level of healthcare institutions, which partly explains why it is also the group most present in the scientific research of the sample analysed in this manuscript. However, according to the WHO and the Sustainable Development Goals of the 2030 Agenda for Sustainable Development (SDGs) [40], the nursing workforce is insufficient and unevenly distributed around the world. The countries with the greatest shortages are low-income and lower-middle-income countries, where the growth of nurses is not keeping pace with population growth. On the other hand, increasing longevity in Western countries and low birth rates portend a near future of an ageing population in need of more healthcare. Approximately 90% of the nursing staff are women, although management positions do not have the same ratio. Although it is not the purpose of this research, it should be noted that the report on “The State of the World’s Nursing 2020” prepared by the WHO [39] offers recommendations to address various problems and concerns that have been reflected in the literature review provided in this research.

### 1.2. Organisational Commitment and Healthcare

Organisational commitment was defined by Porter and Lawer [41] as the desire developed by the employee that leads him/her to make high efforts for the good of the institution, to long to remain in it, and to accept its main objectives and values. A revision of this definition was made by Greenberg and Baron [42] where they indicate that organisational commitment is the level of identification that employees feel with the organisation where they work, which conditions the degree of commitment they show and the willingness to leave it.

Organisational commitment can be distinguished under three different perspectives. At the end of the 20th century, Meller and Allen [43,44] developed the social exchange perspective described by Becker in 1960 [45]; the authors describe what they call continuity or permanence commitment, which is based on the small investments that the worker has developed over time and which slow down his or her possible voluntary disengagement from the organisation. In healthcare, continuity commitment appears in older and more experienced nurses, who are more willing to remain in the job [46,47,48], although it also depends on the existence of other job opportunities in their environment [49].

From a psychological orientation, we find affective commitment [50,51], which is characterised by the desire shown by the employee to remain a member of the organisation, identifying with and accepting its values and goals in exchange for obtaining certain psychological rewards, such as recognition or the support of his or her team. Within the healthcare setting, affective commitment has been positively related to nurses’ job satisfaction and intention to stay in the job [16,49]. Likewise, the team structure and networking behaviour of the organisation with the employee has shown a positive link with affective commitment [52]. As in all other organisations, affective commitment is strongly related to trust in the organisation, which is very much present in the employee–employer healthcare system.

Meyer and Allen [44], develop the so-called normative commitment, which is more related to the work ethic and the responsibility that the worker acquires and imposes on himself in his job. This encourages him to do his job to the best of his ability at all times, even if he does not agree with the characteristics of his job or the organisation to which he is attached. Normative commitment has been widely discussed in the scientific literature on healthcare [25,53,54], and multiple research and case studies indicate that normative commitment depends on a large number of factors such as gender, age of the worker, place of birth, work context, type of institution, etc.

Healthcare organisations often encourage continuity or tenure commitment, for the reasons of scarcity discussed above, by offering high salaries and attractive benefits, which may mask the levels of affective and normative commitment of the healthcare workforce [55]. In healthcare settings, both the mistakes that are made and the consequences associated with these mistakes could be minimised with high organisational commitment on the part of employees, which is not always achieved [56].

### 1.3. Rationale, Objectives, and Practical Implications

Research on human resource management in healthcare institutions is booming. The leaders and managers of these institutions face numerous challenges in order to achieve an effective and efficient organisation, for which the organisational commitment of their employees is an indispensable tool for success. The numerous research studies on the management of hospital staff describe a large number of case studies on this issue. These studies are related to healthcare institutions of all kinds, which are located in many different countries and focus on a wide variety of problems in these institutions. However, a number of common features can be seen in these studies, such as shortage of nursing staff, high turnover, employee burnout, the intention to leave the job or the profession, etc. This set of situations is closely linked to the absence of job satisfaction, fulfilment of the psychological contract, and organisational commitment.

Proof of the importance of the topics mentioned above is the existence of a large number of journals specifically dedicated to the management and human resources of health centres and more specifically to nursing: *Journal of Nursing Management* [57], *Journal of Advanced Nursing* [58], *Health Care Management Review* [59], *Human Resources for Health* [60], or *International Journal of Health Planning and Management* [61].

Given the justified need to improve the management of human resources in health institutions, the main objective of this research is to broaden and improve knowledge about organisational commitment in the health sector, in order to explore the factors that have an impact on this subject and how they are related. The results of this work will allow, in addition to exposing the most important issues of this subject, to detect those areas of research that have not been sufficiently developed, creating points of support for future research, and at the same time, these results may help administrators, managers, and human resources managers of health centres make decisions about their human resources strategy. For this purpose, a study of the activity indicators of the published literature is carried out by means of a descriptive statistical analysis, detailing which authors and journals have been the most prolific in this area. Furthermore, by means of bibliometric techniques, we describe which topics are the most prominent in the literature related to organisational commitment in the healthcare field, where a longitudinal analysis of the research currents on these aspects is carried out.

The document consists of four parts: (a) Introduction, where the unique characteristics of human resources management in healthcare centres and the importance of organisational commitment are presented; (b) Methodology and materials used; (c) Results, quantitative analysis of the literature, keywords, longitudinal analysis, strategic maps by publication date, and the evolution of research on this topic; and (d) Discussion and conclusions.

## 2. Materials and Methods

The purpose of this research is to analyse research trends regarding organisational engagement and healthcare Institutions. The bibliometric techniques used allow the published material to be examined objectively by organising the information contained in each document [62]. The bibliography consulted that makes up the sample selected in this research and which is detailed in the following sections brings together the main research topics in this field; a metric analysis will allow, among other functions, determining the relationship between them and their evolution over time [63,64]. Ultimately, the aim is to generate useful information for the scientific community as well as for other interest groups related to healthcare institutions and human resources management.

### 2.1. Materials

The documents collected for the review of research on organisational commitment and health institutions came from the Web of Science (WoS) database [65]. The search was conducted on 20 December 2020. The parameters used were “organisational commitment” or “organizational commitment” and “health institutions” or “health system” or “healthcare” or “health care” or “health”. The fields used for the search were: Article Title, Author Keywords, Keywords Plus, and Abstract. The search was restricted to the following indexes: Science Citation Index Expanded (SCI-EXPANDED); Social Sciences Citation Index (SSCI); Emerging Sources Citation Index (ESCI); Art & Humanities Citation Index (A&HCI); Conference Proceedings Citation Index—Social Science & Humanities (CPCI-SSH); Book Citation Index (BKCI); and Science Citation Index Expanded (CCR-EXPANDED). In order to have the largest number of publications associated with this theme, the year of publication was not limited. Likewise, the number of citations received was not restricted, as in some cases, the most recent research has not yet achieved the corresponding scientific impact.

The search obtained a total of 482 manuscripts from WoS. After this first selection, the authors proceeded to identify which articles did not correspond to the purpose of this research. After reviewing the title and abstract of each item, and after a joint assessment, a total of 34 documents were rejected because they were not directly related to organisational commitment and healthcare institutions. The sample finally selected for this bibliometric analysis consists of 448 research articles.

In order to conduct a longitudinal analysis of the literature that has investigated organisational commitment and healthcare institutions, we divided the 448 papers selected from the sample into three periods according to their date of publication. The first article in the sample dates from 1992 and the most recent is from 2020. The criterion for defining the width of each period should correspond to an equivalent number of years; however, the size of the scientific production in the last decade (2011–2020) is much larger than that recorded between 1992 and 2010. Therefore, the choice of the three periods also takes into account the number of manuscripts published at each point in time. Thus, in order to be able to compare periods with a similar and enough publications, the first period chosen is from 1992 to 2010, when a total of 128 documents related to this subject were published in WoS. The second period is defined as between 2011 and 2015, with a total of 131 documents registered. The third and final period runs from 2016 to 2020, with 189 papers present (Table 1).

### 2.2. Software

The bibliometric analysis presented in this document includes the elaboration of longitudinal and strategic maps as well as the construction of thematic networks. The SciMAT software has been used for this purpose. This software synthesises in a single computer package most of the tools used in scientific mapping and also allows the creation of longitudinal maps, which is one of the main objectives of this work. The information provided by the WoS database has been imported into the software used, and we have proceeded as follows: the author’s keywords and the keywords from the source represent the basic unit of analysis; co-occurrences have been used to construct the thematic networks. To normalise the network, the equivalence index is used as a measure of similarity; finally, the scientific topic maps and their corresponding networks are based on the simple centre clustering algorithm.

**Figure 1:**
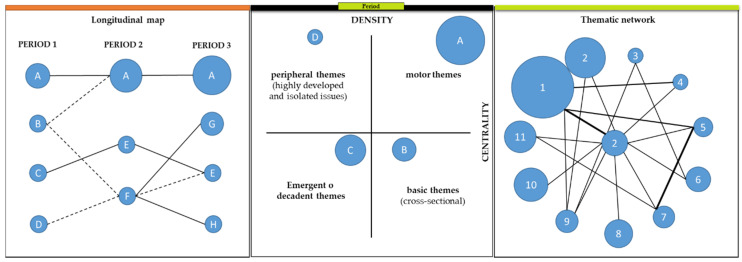
Example of a longitudinal map, strategic map and thematic network. Source: Prepared by the authors of Reference [66].

For a better understanding of the development of this research, we briefly describe the concepts and constructions used. The longitudinal map (Figure 1—left) is composed of as many columns as periods chosen, in our case 3. Each column contains the most relevant themes of that period and the lines that connect with other themes correspond to the evolution of these themes over time. If you want to know what roles the most important themes play in a given period, a strategy map is constructed (Figure 1—centre). In the strategic map, the themes are ordered according to their relevance. The driving themes “A” (Figure 1—centre) are the themes that lead the speciality as they are the best developed and are the most important for the construction of the scientific field. The basic themes “B” (Figure 1—centre) are not yet sufficiently developed, although they make important contributions to the scientific field under study. Emerging or decadent themes “C” (Figure 1—centre) are themes that are underdeveloped and not very significant at the moment; they may become important themes in the future or, on the contrary, disappear. Finally, the peripheral themes “D” (Figure 1—centre) appear as very specialised themes within the subject analysed. From each cluster or main topic shown in the longitudinal (Figure 1—left) and strategic maps (Figure 1—centre), a graph can be extracted called a thematic network (Figure 1—right); this network is formed through the interconnection of the keywords between documents of each topic.

## 3. Results

### 3.1. Activity in the Literature on Organisational Commitment and Healthcare

**Figure 2:**
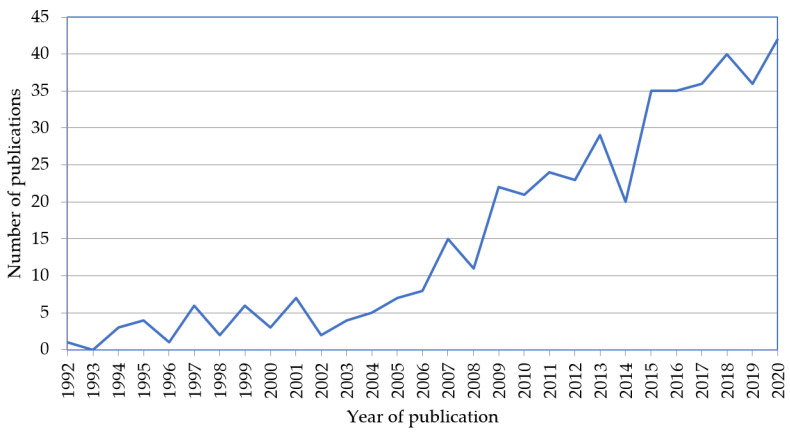
Publications over time on organisational commitment and healthcare (*n* = 448). Source: Prepared by the authors on the basis of Web of Science (WoS) data.

The 448 documents that form the WoS sample for our research were published between 1992 and 2020 (Figure 2). The annual publication rate describes two distinct trends. From 1992 to 2006, scientific production in this field is not very numerous; it is from 2006 onwards, and possibly driven by the 2006 World Health Organisation report [19], that scientific production describes an upward trend that continues to the present day.

The 448 documents in our sample included in the WoS database were written by 1546 authors. Despite the large size of the selected sample, there are no authors with a much higher number of articles than the rest, which gives an idea of the breadth and diversity of areas of knowledge that have addressed organisational commitment in health centres. Table 2 shows the authors who have published the most research, the dates of their first and last work on this topic, and the number of times they have been cited from other publications included in WoS.

From the sample obtained in this research, we can highlight the documents published by Laschinger, HK or Cummings, G, which include literature review articles [67,68,69,70] and works related to management and commitment [49,55], job satisfaction [71], working conditions [7,72,73], and consequences in the field of healthcare institutions. We can also highlight Shamian, J. who, despite having participated in only three articles in this sample, obtained a significant number of citations, where the document “*Nurse turnover: A literature review*” [74], in which Laschinger, HK also participates, stands out. Within the set of selected articles, the following research could be highlighted for the citations received and for being precursors of this topic: in 1992, Richardsen et al. published “*Occupational demands, psychological burnout and anxiety among hospital personnel in Norway*” [75] with 52 citations received; in 1995, Gerhon et al. published “*Compliance with universal precautions among health-care workers at 3 regional hospitals*” [76] with 174 citations received; and also in 1995, Mcneese-Smith published “*Job-satisfaction, productivity, and organisational commitment—the result of leadership*” [23], which was cited 69 times.

The journals with the highest presence of research on organisational commitment and healthcare in the sample are shown in Table 3. The 448 documents analysed were published in 274 different journals. It should be noted that most of the journals that have dealt with the subject matter of this research are related to health sciences; i.e., they are specialised publications in health, nursing, and other healthcare fields, although there are also others related to management

The publications in the sample analysed come from a variety of countries around the world. In fact, most of the articles deal with case studies on health centres and their workers, so that the sample allows us to investigate the different aspects related to human resources in health centres. In order to present in a simple way the countries where scientific production in this field has been most numerous, the residence of the first author has been taken as a reference. This avoids counting errors in cases where articles are presented with several authors of the same nationality, which would lead to an incorrect classification. Of the 53 countries represented, the North American nations stand out above the rest: the USA with 128 manuscripts and Canada with 47. At some distance, Turkey appears with 28, Australia with 20, the UK and China with 18, Iran and the Netherlands with 17, Taiwan with 15, and Finland with 14 articles published in WoS.

Research on Organisational Engagement and Healthcare is characterised by many different authors from all parts of the globe, although with a greater presence in North American countries. The manuscripts are mainly case study oriented and have been published mainly in healthcare-oriented journals. The following is a more detailed analysis of the topics most present in the publications of the sample obtained.

### 3.2. Evolution of Keywords

**Figure 3:**
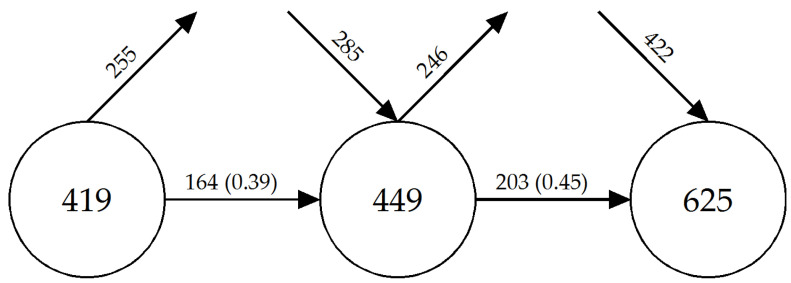
Keywords between periods. Source: Prepared by the authors on the basis of SciMAT data.

In order to find elements that indicate the state of development of a field of research, the use of keywords and how they have evolved over time in the different periods is analysed, using the method of Price and Gürsey [77] (Figure 3). This methodology represents the periods analysed with circles, in which the keywords that have been used in that period are listed inside them. The circles are linked by an arrow representing the keywords that are used from one period to another, and in brackets, the stability index or overlap fraction. The incoming arrows in each circle represent the keywords that are used for the first time, and the outgoing arrows symbolise the keywords that are no longer used in the following period.

With regard to the results of the evolution of the keywords, it should be noted that the first two periods (1992–2010/2011–2015) are characterised by maintaining a certain similarity in the number of keywords: the first period (419) and the second (449). There has barely been an increase of 30 new keywords, which represents a growth of 7%. The stability index between the two periods is 0.39, which is considered relatively low; i.e., just over one-third of the keywords from the first period have been used in the second period. Concerning the transition between the second (449) and third period (625), the growth is much more remarkable. The volume of keywords has increased by 176, which is a growth of 39.2%. The stability index (0.45) between the second and third period was slightly higher than in the previous period, but it was still low. Therefore, there is evidence of a growth of new lines of research in the last period, which is characterised by a clear inconsistency of vocabulary [77]. This situation suggests that this field of research can admit a wide margin for development.

### 3.3. Longitudinal Analysis

**Figure 4:**
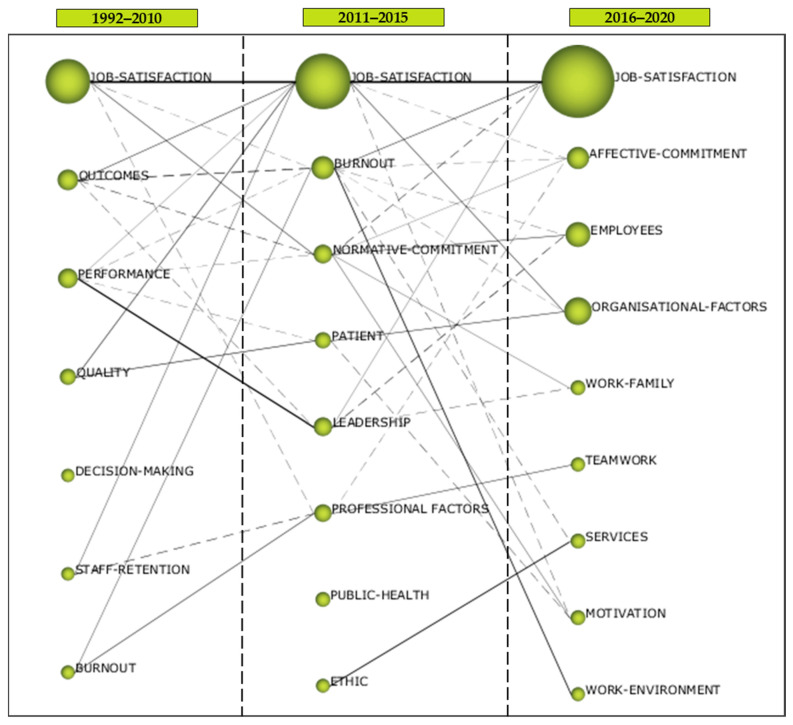
Evolution of the themes of primary documents. Source: Prepared by the authors on the basis of SciMAT data.

Once it has been established that there is a wide margin of development in the literature relating organisational commitment (OC) and healthcare (HC), the next objective is to analyse, by means of longitudinal maps, the evolution of research work related to these two concepts (Figure 4). The first finding, due to its significant consistency, is the persistence of the *Job-satisfaction* cluster as the leader of the research trend throughout the three periods analysed. This cluster, with respect to the others, agglutinates a greater number of documents, maintaining a constant growth period after period (72 docs, 1992–2010; 96 docs, 2011–2015; and 134 docs, 2016–2020) (Table 4). However, the behaviour of the impact of publications on the topics to which this cluster is related has been decreasing. Thus, in the first period, the impact was 6194 citations, in the second period, it was 2322 citations, and in the third period, it was only 870 citations. The explanation for these last two periods may lie in the short time span, and the significant impact of the first may be due to the contribution of various literature reviews and meta-analyses such as the work of Humphrey et al. in 2007 [78], “*Integrating Motivational, Social, And Contextual Work Design Features: A Meta-analytic Of The Summary And Theoretical Extension Work Design Literature*”, with 863 citations; Thoresen et al. in 2003 [79], “*The Affective Underpinnings Of Job Perceptions And Attitudes: A Meta-analytic Review And Integration*”, 458 citations; Hayes et al. in 2006 [74], “*Nurse Turnover: A Literature Review*”, 457 citations; Cheng and Chan in 2008 [47], “*Who Suffers More From Job Insecurity? A Meta-analytic Review*”, with 455 citations; Lu et al. in 2005 [9], “*Job Satisfaction Among Nurses: A Literature Review*”, with 353 citations, among others.

In the longitudinal analysis, all the periods are important because they give us an idea of how the literature has evolved; however, the first one is particularly important, as it brings together the research that initiated the interest in the subject, and the last one, which provides a perspective on the current research interest or trend. In the case of the first period (1992–2010), we find that in addition to *Job-satisfaction*, research originated around the following clusters: *Outcomes*, *Performance*, *Quality*, *Burnout*, *Decision-making,* and *Staff-retention*. From a quantitative point of view (number of papers), the most significant, apart from *Job-satisfaction*, were *Outcomes* and *Performance*. In terms of the impact of the publications, the third and last in the list of clusters with the most documents should be highlighted; *Performance* with 1910 citations and, strikingly, *Staff-retention* with 305 citations in only three documents, including the work of Spence et al. [8], “*Workplace Empowerment, Incivility, And Burnout: Impact On Staff Nurse Recruitment And Retention Outcomes*” with a total of 275 citations. As for the last period (2016–2020), which concentrates the topics with the highest current trend, we find (ordered by highest number of documents) in addition to *Job-satisfaction*, *Organisational-factors*, *Employees*, *Affective-commitment*, *Work-family*, *Services*, *Motivation*, *Teamwork,* and *Work-environnment*. The most relevant clusters in terms of the impact of their publications are *Job-satisfaction* (870 citations), *Employees* (242 citations), *Organisational-factors* (219 citations), and *Affective-commitment* (122 citations).

From the point of view of the evolution of the clusters between periods, it should be noted that the only cluster that is maintained in the three periods and, as a result, retains its consistency is the aforementioned *Job-satisfaction* which, in addition, has been the recipient in the second period (2011–2015) of themes from the first (1992–2010) such as *Outcomes*, *Staff-retention*, *Performance,* and *Quality*. Although some of the themes of the latter two clusters have also evolved towards *Leadership* and *Patient*, respectively. As for the *Burnout* cluster, it is the only cluster that has been maintained for two consecutive periods, from the first to the second. Although it is true that some of its themes from the first period also evolved towards the *Professional factors* cluster.

By way of conclusion, it can be suggested that (a) the motivation for research on OC and HC in a related way started on clusters that brought together topics that had to do with job satisfaction, analysis of results, performance, quality, and burnout syndrome, mainly; (b) only two clusters have remained in more than one period over time, *Job-satisfaction,* which has remained in all three periods, and *Burnout,* which has remained between the first (1992–2010) and the second period (2011–2015), while the rest of the clusters have dispersed between the periods; and (c) research is currently focussed, in addition to job satisfaction (which has been the same since the beginning), on issues related to organisational factors, on aspects related to employees such as leadership or attitudes, on affective commitment and, to a lesser extent, on aspects such as work–life balance, motivation or work climate.

### 3.4. Period-by-Period Strategy Map Analysis

**Figure 5:**
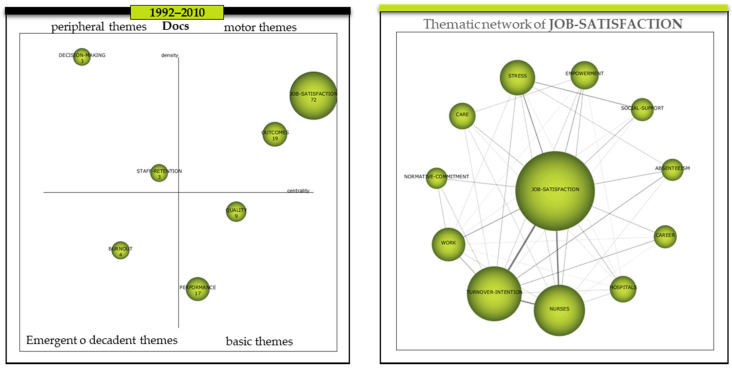
Evolution of the themes period 1992–2010 and thematic network of the cluster JOB-SATISFACTION. Source: Prepared by the authors on the basis of SciMAT data.

Once we know, through the longitudinal analysis, the evolution of the clusters or themes between periods, we first proceed to analyse the importance of each cluster in the field of research. For this, we use the “Strategy Maps” (Figure 5-left). These provide an overview of the role played by the clusters in terms of their centrality and density. Secondly, we are interested in finding out about the main theme, i.e., the cluster which, due to its centrality and density, occupies the top right-hand position on the strategy map, which the methodology used calls “Driving Themes”. The next step is to analyse the structure of its network, in order to find out which themes have been researched and what the relationship between them has been. This is the essential core of the literature review in terms of finding out the trend of researchers’ interest. In short, it is about finding the main driving theme and then analysing how researchers have become interested in the themes of this cluster in a related way. For this, we use the “Thematic network” maps (Figure 5—right).

In the period 1992–2010, we mainly found two driving themes (Figure 5-left), *Job-satisfaction* and *Outcomes*, with a centrality and density of 114.71/29.63 and 68.79/19.50, respectively (Table 4). Therefore, we can confirm that they were the trending topics or clusters in this period, i.e., the ones that had the ability to attract more publications around them, in a related way, with other topics in their internal network. They were at the centre of the research. As core topics, i.e., those that are important for the research field, but need to be developed, we find *Quality* and *Performance*, whose centrality and density were 35.26/12.09 and 30.11/9.54 respectively. As for the more specialised topics, which the methodology used calls “peripheral”, we find *Decision-making* and *Staff-retention*. These have a high density but not sufficient centrality. In the case of the two clusters mentioned above, their centrality and density were 6.14/50 and 11.48/16.67, respectively. Finally, in the lower left quadrant, there are those clusters that due to their low centrality and density may be in a situation of decay or emergence; in this period, the *Burnout* cluster is in this position with a centrality and density of 6.24/11.30.

In order to go into the internal analysis of the thematic network of the main driving theme of each period, in order to be able to compare the different periods, we have analysed the dyads of those relationships that exceed a minimum weight limit of 0.10 or more.

In the case of the first period (1992–2010), in the internal analysis of the *Job-satisfaction* thematic network, the main driving theme and, as a consequence, the cluster leading the research trend, we found with a density equal to or greater than 0.10 the following dyads (Table 5): (a) *Job-satisfaction* with the themes *Turnover-intention*, *Nurses*, *Stress*, *Work*, *Empowerment,* and *Absenteeism*, (b) *Nurses* with *Turnover-intention*, (c) *Stress* with *Social-support*, (d) *Turnover-intention* with the themes *Absenteeism*, *Work,* and *Career*. The themes that—although important as a trend—did not reach the established limit of 0.10 weight in the density of their relationships within the cluster were *Hospitals*, *Care*, and *Normative commitment*.

**Figure 6:**
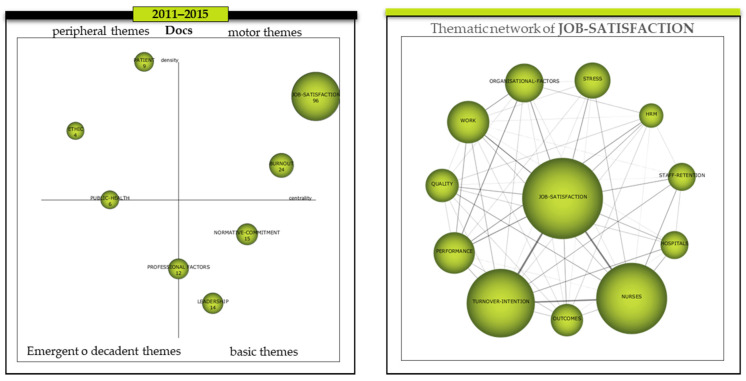
Evolution of the themes period 2011–2015 and thematic network of the cluster JOB-SATISFACTION. Source: Prepared by the authors on the basis of SciMAT data.

As for the period 2011–2015, *Job-satisfaction* repeats as the main driving theme with a centrality and density of 144.69 and 43.25 respectively, which is even higher than in the previous period (Table 4). This is joined by *Burnout*, which evolves from the previous period, when it was considered as a declining or emerging theme (Figure 6-left). Its centrality and density were 53.12 and 16.5, which was much higher than in the previous period. As basic themes, we find *Normative-commitment* and *Leadership*, whose centrality and density were 47.56/14.79 and 43.68/9.97, respectively. On the other hand, on the border between the emerging themes and the basic themes is the *Professional-factor* cluster, with a centrality of 27.07 and a density of 14.65, and, on the border between these and the peripheral themes, the *Public-health* cluster with a centrality of 9.3 and a density of 15.48. Finally, in the upper left quadrant, the peripheral themes formed in this period are *Patient* and *Ethic* with a centrality and density of 9.3/15.48 and 4.65/22.22, respectively.

With regard to the internal analysis of the thematic network of the main driving theme, *Job-satisfaction* (Figure 6-right), it should be noted that in general, the density of the relationships of the members of the thematic network has been higher. Proof of this is that the weight of the ties of the dyads that relate the *Job-satisfaction* theme to the rest of the themes was equal to or greater than 0.10 (Table 6); however, this was not the case in any of the previous periods. As for the rest of the dyads that equalled or exceeded 0.10 weight in their relationships, the following should be noted: (a) *Nurses* with the themes *Turnover-intention*, *Staff-retention*, *Quality,* and *Hospitals*, (b) *Performance* with the themes *Organisational-factors*, *HRM,* and *Work*, (c) *Work* with themes such as *Organisational-factors* and *Turnover-intention*, (d) *Hospitals* with *Turnover-intention,* and finally, (e) *Turnover-intention* with *Staff-retention*. The themes that formed part of this cluster, but with a density of less than 0.10 in the relationships other than the main theme (*Job-satisfaction*), were *Stress* and *Outcomes*.

**Figure 7:**
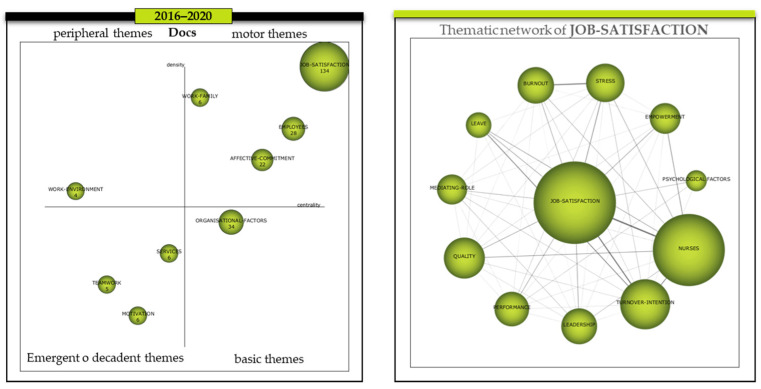
Evolution of the themes period 2016–2020 and thematic network of JOB-SATISFACTION. Source: Prepared by the authors on the basis of SciMAT data.

Finally, in the 2016–2020 period, *Job-satisfaction* maintains its leadership as a reference cluster for the research trend concerning the literature relating OC and HC and, although its centrality (108.78) and density (28.93) have decreased with respect to the two preceding periods (Table 4), compared to the rest of the clusters in this period, it has achieved an even more prominent position (top right position) (Figure 7). The other clusters that form part of the so-called “Driving Themes” are *Employees*, *Affective-commitment,* and *Work-family* with a centrality and density of 53.17/10.87, 52.76/10.21, and 14.89/19.44, respectively. In the lower right quadrant, only *Organisational-factors*, which has a centrality of 44.93 and a density of 8.34, is found as a basic theme. There is also only one theme in the so-called “peripheral themes”, *Work-environment*, with a centrality of 7.14 and a density of 9.88 and, finally, in the emerging or declining themes, there are *Services*, *Motivation,* and *Teamwork*, with a centrality and density of 14.14/8.30, 10.18/3.76, and 8.17/5.36 respectively.

With regard to the analysis of the *Job-satisfaction* thematic network, as the main driving theme, we find a less dense network than in the previous period. The dyads that equal or exceed a weight above the stipulated 0.10 are (Table 7) (a) *Job-satisfaction* with the themes *Nurses*, *Turnover-intention*, *Stress*, *Leadership*, *Leave,* and *Empowerment*, (b) *Burnout* with *Stress*, (c) *Nurses* with themes such as *Turnover-intention*, *Empowerment*, *Quality,* and *Leave* and, finally, (d) *Turnover-intention* with *Leave*. The themes that do not have a density equal to or greater than 0.10 were *Mediating-role*, *Psychological factors,* and *Performance*.

From a cross-sectional perspective, it is evident that the main driving theme has consistently been *Job-satisfaction*, which means that the research trend related to organisational commitment and the healthcare system has been oriented towards the themes integrated in the network of this cluster. Analysing their behaviour longitudinally, we can see that the themes that have permanently belonged to this cluster throughout the three periods have been *Job-satisfaction*, *Nurses*, *Turnover-intention,* and *Stress*, and therefore, due to their relational solidity, they could be considered to be the main core of the research. It should be noted that themes such as *Performance* and *Quality*, which in the first period (1992–2010) were considered basic themes, i.e., not very developed, were incorporated in the second period (2011–2015) into the cluster of the main driving theme (*Job-satisfaction*) and have been maintained in the third period (2016–2020). Something similar happens to *Burnout*, which in the first period formed an independent cluster as an emerging or declining theme and, in the second period, this cluster evolved with greater centrality and density in its network, being classified as a driving theme. Finally, in the third period, *Burnout* is incorporated into the *Job-satisfaction* cluster. On the other hand, *Empowerment*, which already belonged to the *Job-satisfaction* cluster in the first period, was reincorporated from the first to the third period. Finally, the most relevant themes that have been incorporated for the first time in the third period, apart from *Burnout*, are *Leave*, *Psychological factors,* and *Leadership* (Figure 5—right); (Figure 6—right); (Figure 7—right).

## 4. Discussion

From a longitudinal perspective, the aim of this work has been to broaden knowledge about the literature relating organisational commitment and healthcare, not only from the point of view of the evolution of the subject in a related way, but also by focussing on those topics that are not sufficiently developed. On the other hand, the idea of this research was not to go into the depth of the content. Rather, the aim has been to shed light on a research horizon on organisational commitment in complex health system settings and to create points of support for future research, as well as to serve as an aid to decision making in health organisational settings. The methodology used is relevant and effective, and it yields results in a stepwise approach in which layers of knowledge are uncovered, both in terms of research output and the most influential issues in the field under investigation. This methodological approach also provides information on the degree of maturity or saturation of the subject matter under study, where research is heading, and whose areas have not yet been addressed.

Looking at the analysis of the literature that has investigated organisational commitment in healthcare institutions, the results unequivocally suggest that the most prolific and centralised line of research over time has been that which has analysed organisational factors related to nurses. These have focussed mainly on aspects related to job satisfaction, including the implications of stress and high turnover, burnout syndrome, psychosocial factors, and the possibility of leaving the job. Of particular interest in the latter period has been research on the impact of empowerment in the workplace and its relationship with leadership and job satisfaction. Finally, research on performance and service quality, aspects oriented towards organisational management and closely related to job satisfaction and commitment, has been growing in interest over time.

Another of the interesting findings has been to ascertain the accentuated research orientation of the scientific community in the investigation of problems in the HRM of nursing staff, with respect to the rest of the groups with which they coexist in healthcare organisations. In this sense, the literature has shown sufficient reasons to consider this inclination justified; however, the low interest in the rest of the groups such as doctors, specialists, auxiliary staff, or administrative structure is not justified. An example of this is that barely twenty documents in the sample of this work have shown interest in some aspect related to doctors.

On the other hand, the results of this research have highlighted the lack of studies that delve deeper into issues such as the psychological contract and its relationship with organisational commitment in the healthcare context, or the importance of normative commitment within the types of organisational commitment in the healthcare sector. In this sense, there is research suggesting that normative commitment, together with increasing quality at work, are good strategies to reduce staff turnover intentions [54]. The literature has also shown that breaching the psychological contract affects organisational commitment [31,32,44,80,81,82,83]. Another connection of the psychological contract with organisational commitment concerns the so-called ideologically charged psychological contracts [29,84,85,86]. This is ideologically inspired employee behaviour. This approach suggests that the employee recognises his or her contribution to the achievement of a greater good aligned with the organisation’s goals. He understands that the achievement itself provides him with intrinsic motivation and this encourages him to continue to cooperate with the organisation in the belief that it is the right thing to do. Along these lines, Meyer and Parfyonova [83] attempt to relate the ideological psychological contract to normative commitment when there are ethical underpinnings. The literature has also related ideological psychological contracts and normative commitment to the public sector and to organisations with a significant moral or ethical burden, such as the health sector. In this sense, research linking organisational commitment and, more specifically, normative organisational commitment, in relation to the fulfilment of the ideological psychological contract in the context of healthcare organisations, is important.

### Limitations

In this paper, the authors recognise that the research has certain limitations. On the one hand, that which comes from using documents exclusively from the Web of Science (Wos) database. On the other hand, there are the limitations that arise when applying filters to achieve the necessary precision in the search for published research.

## 5. Conclusions

The complexity and mix of effectiveness and efficiency objectives in healthcare organisations, together with the high ethical and moral standards expected, the continuous technological change, clinical advances, or unexpected global health challenges such as the management of pandemics, make the management of human resources in this type of organisations a real challenge. Therefore, if in general, business organisations are in need of professionals who feel committed to the strategic objectives of the organisation, in the field of health, the need is even greater. In this sense, the aim of this paper has focussed on shedding light on the literature that investigates organisational commitment in the health sector. To this end, bibliometric techniques have been used on a sample of 448 research studies published in the Web of Science database between 1992 and 2020.

Moving on to the analysis of the results, with regard to the evolution of research production related to the subject under study, it should be noted that there is a period between 1992 and 2006 in which the number of publications is relatively low; however, from 2006 until 2020, probably due to the WHO report of 2006 [39], the number of publications rises notably. From the point of view of the number of articles per author, the production in the literature is homogeneous; however, Cummings, G. stands out for the volume of publications and Laschinger, H. K. and Shamian, J. stand out for the number of citations. The countries with the highest scientific production on this subject are the USA and Canada. In relation to the margin of development of the subject, the sustained growth of research production, together with the high rate of incorporation of keywords and a low rate of stability between periods, suggest that the field of research is of high interest to researchers and maintains a wide margin of development. As for the analysis of the evolution of the subject matter, a set of topics that have been researched in a related way over time and that form part of the main driving theme of each period analysed is evident. These form the main core of the research on organisational commitment in the field of health and revolve around *Job-satisfaction*, *Nurses*, *Turnover-intention*, *Stress*, *Burnout*, *Psychological factors*, *Leave*, *Empowerment,* and *Quality*. In terms of possible trends that are consolidating in the last period, themes such as *Work–family*, *Affective-commitment,* and *Organisational-factors* stand out. In terms of areas that have not yet been sufficiently developed or are not yet present in the literature of the sample analysed, and which should be the subject of future research work, on the one hand, there is the analysis of the organisational commitment of other professional profiles within the field of health organisations, such as doctors, specialists, auxiliary staff, or administrative management structure; on the other hand, there is a need to explore the impact of the ideological psychological contract on normative organisational commitment, which are aspects that the recent literature considers to be particularly related to the public sphere and in particular to the health sector, and which need to be developed further.

## Figures and Tables

**Table 1:** Periods and number of documents per period.

Number	Period	No. of Documents
1	1992–2010	128
2	2011–2015	131
3	2016–2020	189

Source: Prepared by the authors based on SciMAT data.

**Table 2:** Authors who have published three or more articles on organisational commitment and healthcare.

Author	Number of Articles	Year of Publication First and Last Article	Cited by
Cummings, Greta	9	2009–2018	270
Laschinger, Heather K.	7	1999–2015	985
Boston, C	4	1994–1995	9
Wong, Carol A.	3	2013–2018	135
While, Alison E.	3	2007–2012	364
Top, Mehmet	3	2012–2015	78
Shamian, J	3	2000–2006	640
Rodwell, J	3	2009–2015	31
Rathert, Cheryl	3	2009–2015	49
Portoghese, Igor	3	2012–2018	5
O’Keeffe, Daniel F.	3	2015–2015	29
Macinati, Manuela S.	3	2014–2020	12
Lu, Hong	3	2007–2012	364
Liou, Shwu–Ru	3	2008–2013	19
Leggat, Sandra G.	3	2007–2012	152
Kovner, Christine T.	3	2009–2015	245
Galletta, Maura	3	2012–2018	5
Fitzpatrick, Joyce J.	3	2009–2016	88
Demir, Defne	3	2009–2015	31
Clausen, Thomas	3	2015–2018	14
Cheng, Ching–Yu	3	2008–2013	19
Brewer, Carol S.	3	2009–2015	245
Barriball, K. Louise	3	2007–2012	364

Source: Prepared by the authors the basis of WoS data.

**Table 3:** Journals that have published on organisational commitment and healthcare. Quartile 2019 and total items.

N.	Journal	Q1	Q2	Q3	Q4	Total Items	%	First Doc.	Last Doc.
1	*Journal of Nursing Management*	X	-	-	-	23	5.1%	2009	2020
2	*Journal of Advanced Nursing*	X	-	-	-	16	3.6%	2006	2020
3	*Health Care Management Review*	X	-	-	-	16	3.6%	2005	2020
4	*Journal of Nursing Administration*	-	-	X	-	15	3.3%	1994	2020
5	*International Journal of Nursing Studies*	X	-	-	-	13	2.9%	2005	2019
6	*Int. Journal of Human Resource Management*	-	X	-	-	10	2.2%	2001	2020
7	*BMC Health Services Research*	-	-	X	-	10	2.2%	2007	2018
8	*Human Resources for Health*	X	-	-	-	8	1.8%	2013	2020
9	*Personnel Review*	-	X	-	-	7	1.6%	2011	2020
10	*Nursing Ethics*	X	-	-	-	7	1.6%	2012	2019

Source: Prepared by the authors on the basis of WoS data. Q1, Q2, Q3, and Q4 correspond to the quartile of Journal Citation Report (2019).

**Table 4:** Quantitative and qualitative factors of the themes and their evolution.

	1992–2010					2011–2015					2016–2020				
Name	Centrality	Density	Documents	Citations	H-Index	Centrality	Density	Documents	Citations	H-Index	Centrality	Density	Documents	Citations	H-Index
Job-satisfaction	114.71	29.63	72	6194	41	144.69	43.25	96	2322	28	108.78	28.93	134	870	15
Outcomes	68.79	19.50	19	902	15	-	-	-	-	-	-	-	-	-	-
Performance	30.11	9.54	17	1910	16	-	-	-	-	-	-	-	-	-	-
Quality	35.26	12.09	9	354	8	-	-	-	-	-	-	-	-	-	-
Burnout	6.24	11.30	4	385	4	53.12	16.56	24	689	15	-	-	-	-	-
Decision-making	6.14	50.00	3	93	3	-	-	-	-	-	-	-	-	-	-
Staff-retention	11.48	16.67	3	305	3	-	-	-	-	-	-	-	-	-	-
Normative commitment	-	-	-	-	-	47.56	14.79	15	258	9	-	-	-	-	-
Leadership	-	-	-	-	-	43.68	9.97	14	417	10	-	-	-	-	-
Professional factors	-	-	-	-	-	27.07	14.65	12	480	9	-	-	-	-	-
Patient	-	-	-	-	-	14.49	162.60	9	139	6	-	-	-	-	-
Public-health	-	-	-	-	-	9.30	15.48	6	111	4	-	-	-	-	-
Ethic	-	-	-	-	-	4.65	22.22	4	66	4	-	-	-	-	-
Organisational-factors	-	-	-	-	-	-	-	-	-	-	44.93	8.34	34	219	8
Employees	-	-	-	-	-	-	-	-	-	-	53.17	10.87	28	242	8
Affective commitment	-	-	-	-	-	-	-	-	-	-	52.76	10.21	22	122	7
Work–family	-	-	-	-	-	-	-	-	-	-	14.89	19.44	6	44	3
Services	-	-	-	-	-	-	-	-	-	-	14.14	8.30	6	24	2
Motivation	-	-	-	-	-	-	-	-	-	-	10.18	3.76	6	38	4
Teamwork	-	-	-	-	-	-	-	-	-	-	8.17	5.36	5	16	3
Work-environment	-	-	-	-	-	-	-	-	-	-	7.14	9.88	4	23	3

Source: Prepared by the authors on the basis of SciMAT data.

**Table 5:** Thematic network JOB-SATISFACTION 1992–2010. Relationships with a weight equal to or greater than 0.10.

N.	Node A	Node B	Weight
1	Job-satisfaction	Turnover-intention	0.46
2	Job-satisfaction	Nurses	0.36
3	Nurses	Turnover-intention	0.30
4	Job-satisfaction	Stress	0.18
5	Job-satisfaction	Work	0.14
6	Stress	Social-support	0.14
7	Turnover-intention	Absenteeism	0.12
8	Job-satisfaction	Empowerment	0.12
9	Turnover-intention	Work	0.11
10	Career	Turnover-intention	0.10
11	Job-satisfaction	Absenteeism	0.10

Source: Prepared by the authors on the basis of SciMAT data.

**Table 6:** Thematic network JOB-SATISFACTION 2011–2015. Relationships with a weight equal to or greater than 0.10.

N.	Node A	Node B	Weight
1	Job-satisfaction	Turnover-intention	0.44
2	Job-satisfaction	Nurses	0.38
3	Nurses	Turnover-intention	0.33
4	Job-satisfaction	Work	0.21
5	Job-satisfaction	Performance	0.18
6	Performance	Organisational-factors	0.17
7	Job-satisfaction	Organisational-factors	0.16
8	Job-satisfaction	Stress	0.14
9	Performance	HRM	0.14
10	Work	Organisational-factors	0.14
11	Hospitals	Turnover-intention	0.13
12	Nurses	Staff-retention	0.13
13	Job-satisfaction	Staff-retention	0.13
14	Nurses	Quality	0.12
15	Job-satisfaction	Quality	0.12
16	Performance	Work	0.12
17	Job-satisfaction	Hospitals	0.11
18	Job-satisfaction	Outcomes	0.11
19	Turnover-intention	Staff-retention	0.11
20	Hospitals	Nurses	0.10
21	Turnover-intention	Work	0.10
22	Job-satisfaction	HRM	0.10

Source: Prepared by the authors on the basis of SciMAT data.

**Table 7:** Thematic network JOB-SATISFACTION 2016–2020. Relationships with a weight equal to or greater than 0.10.

N.	Node A	Node B	Weight
1	Job-satisfaction	Nurses	0.34
2	Job-satisfaction	Turnover-intention	0.26
3	Burnout	Stress	0.24
4	Nurses	Turnover-intention	0.18
5	Turnover-intention	Leave	0.17
6	Nurses	Empowerment	0.14
7	Job-satisfaction	Stress	0.14
8	Nurses	Quality	0.11
9	Job-satisfaction	Leadership	0.11
10	Nurses	Leave	0.10
11	Job-satisfaction	Leave	0.10
12	Job-satisfaction	Empowerment	0.10

Source: Prepared by the authors on the basis of SciMAT data.

## Data Availability

Not applicable.
